# Women Caring for Children in “the Floating World”

**DOI:** 10.3201/eid1211.AC1211

**Published:** 2006-11

**Authors:** Polyxeni Potter

**Affiliations:** * Centers for Disease Control and Prevention, Atlanta, Georgia, USA

**Keywords:** women and children, cover story, about the cover, Cassatt, Degas,

**Figure:**
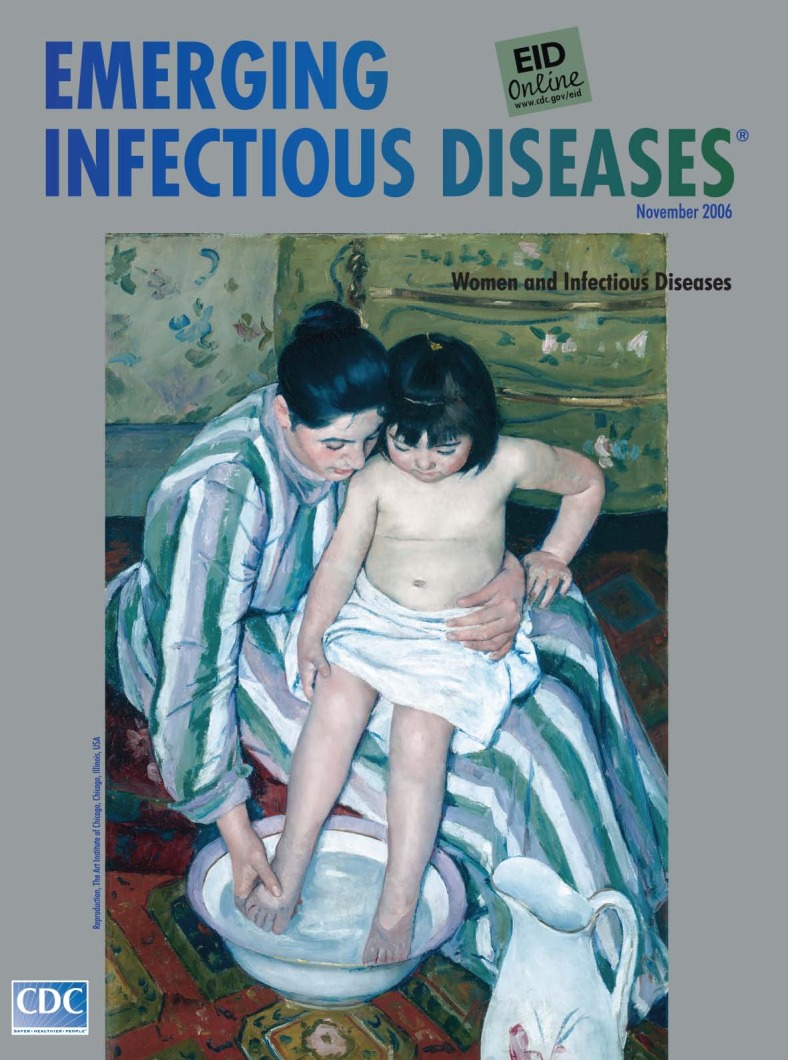
**Mary Cassatt (1844–1926)**. The Child’s Bath (1893). Oil on canvas (100.3 cm × 66.1 cm). Robert A. Waller Fund, 1910.2. Reproduction, The Art Institute of Chicago, Chicago, Illinois, USA

"A figure of a woman is lining up a billiard shot, while the figure of a man…stands dreaming in a doorway," writes artist-printmaker Peter Milton (b. 1930). He is describing the photograph by Gertrude Kasebier in 1908, which inspired his print Mary's Turn. "…it was the drama of the purposeful woman and the pensive man which established the direction Mary's Turn was to take" (*1*). The pensive man was Edgar Degas; the purposeful woman none other than Mary Cassatt, grand dame of impressionism.

Born to an affluent Pennsylvania family, Cassatt enjoyed a privileged childhood and cultural opportunities at home and abroad. Independent and strong willed, she prevailed upon her reluctant parents to let her enroll, at age 16, in the Pennsylvania Academy of Fine Arts in Philadelphia. Against convention, she soon resolved to pursue an artistic career and set off to Paris to study the Old Masters. When the Franco-Prussian War broke out in 1870, she returned briefly to the United States but left again to travel in Italy, Spain, and Belgium and to finally settle in Paris, where she lived the rest of her life.

In the late 1800s, Paris was the center of the art world. Its architecture and transportation system set the standards for 20th-century urban living. Émile Zola described the travails of common people, Claude Debussy found new musical expressions, and French politics was undergoing a democratic revolution. Cassatt set up her studio and studied with academic painter Jean-Léon Gérôme. Her early work was influenced by the realism of Gustave Courbet and Eduard Manet (*2*). She sent her work to the Salon, an annual showcase that judged art on its adherence to agreed upon subjects and strict rules. In 1868, her painting The Mandolin Player was accepted.

The rigid rules of the Salon and passion for creative freedom drove artists to independent exhibits. "I only began to live," Cassatt asserted, "…at the moment Degas persuaded me to… exhibit with his friends in the group of Impressionists. I accepted with joy….I hated conventional art" (*3*). The only American painter to do so, she exhibited often with the impressionists, and under their influence she revised her approach to composition, color, and light, showing admiration for the group, especially Degas.

"It's true. There is someone who feels as I do," Degas once exclaimed in front of one of Cassatt's paintings (*4*). On her part, Cassatt maintained that the first sight of Degas' pastels "was the turning point in my artistic life" (*4*). The two became lifelong friends, supported and influenced each other, and painted portraits of each other. "Oh, my dear, he is dreadful!" Cassatt once confided to her friend, art patron Louisine Havemeyer. "He dissolves all your will power" (*1*). Degas' cantankerous behavior eventually ended the friendship, even though in their old age, both produced great work until both went blind and became unable to paint.

"It is essential to do the same subject over and over again, ten times, a hundred times," advised Degas (*5*). Such intensive involvement with a subject also marked Cassatt's work. Her models were family and friends sitting in the loge at the opera, taking tea, reading, knitting. Over 6 years, she painted more than 20 works exploring the lives of women and their close relationships with children.

"You who want to make color prints wouldn't dream of anything more beautiful….You must see the Japanese," wrote Cassatt to fellow impressionist Berthe Morisot, after visiting an exhibition at the École des Beaux-Arts (*6*). Woodblock prints by such masters as Kitagawa Utamaro and Katsushika Hokusai provided unprecedented views of traditional ukiyo-e, scenes of the floating world (everyday life). Their directness, linear elegance, compositional strength, and tonal richness so impressed and inspired Cassatt that she turned to printmaking. She invented her own techniques and adopted Japanese aesthetics to convey the private mood and intimacy of her domestic scenes.

"I suppose it is…Palmer's French blood which gives her organizing powers and determination that women should be someone and not something," reflected Cassatt about the exceptional qualities of Bertha Honoré Palmer, business woman and philanthropist (*7*), who invited her to paint the south tympanum in the Women's Building at the World's Columbian Exposition in Chicago. The theme, "Modern Woman," was a tribute to women's education, "Young Women Plucking the Fruits of Knowledge and Science." Cassatt so feared the judgment of Degas lest he "demolish me so completely that I could never pick myself up in time to finish for the exposition" that she did not show him the work in progress. On his own part, Degas said of Modern Woman, "I will not admit a woman can draw like that!" (*1*).

Americans were the first patrons of the impressionists, amassing substantial private and museum collections. Cassatt was a frequent advisor to collectors of both Old Masters and the avant-garde. When Louisine Havemeyer sought advice about a New York exhibition in 1915 showing paintings by Cassatt and Degas, as well as by Holbein, Rembrandt, and Vermeer, she advised, "…put a Vermeer of Delft near the Degas and let the public look first at the one and then at the other. It may give them something to think about" (*1*).

"I doubt if you know the effort it is to paint! The concentration it requires, to compose your picture, the difficulty of posing the models, of choosing the color scheme, of expressing the sentiment and telling your story" (*8*). Cassatt was highly skilled. She preferred to work with unposed models placed in asymmetric settings, seen from unusual vantage points. She flattened forms and perspective, contrasted colors and decorative patterns, and used background to establish spatial relationships and shift the focus of perception.

The Child's Bath, on this month's cover, is characteristic of Cassatt's mature work and elaborates on her preferred theme: women caring for children. Preference for the theme reflects her own affection for children and knowledge of 19th-century child-rearing practices. Several cholera epidemics in the mid-1880s prompted official promotion of regular bathing as prevention against disease. And after 1870, French mothers were encouraged to take care of their own children, instead of employing caretakers, and to use modern hygiene practices (*9*).

Cassatt captures a private moment between a woman and a child. The two are absorbed in a domestic ritual, looking down, heads touching, arms interlocked. Aligned along strong diagonals, chubby legs boldly cross ample striped dress, in sharp contrast with circular shapes: heads, washbasin, pitcher. She gently rubs the small foot with one hand, the other holding the child securely in her lap. Lips are parted imperceptibly. Perhaps she is explaining the reflections inside the washbasin. The tender moment, is punctuated by the surroundings: a painted chest-of-drawers, placing the activity on the floor, from the child's perspective, while we have the oblique view from the top. Flowered wallpaper and portions of decorative carpet define the cropped edges of the composition.

"Even more important than the discovery of Columbus which we are gathered here to celebrate," said Bertha Honoré Palmer in her speech on the opening day of the World's Columbian Exposition in 1893, "is the fact that the general government has just discovered women." Though times have changed, Mrs. Palmer's words still ring true in much of the world. Caregiving and safeguard of the physical and emotional health of children go beyond the hygienic benefits of the bath and are tightly connected with the physical and emotional health of the caregiver. To remedy long neglect of the caregiver and protect against emerging health threats, it is time, as Cassatt put it, for women to pluck the fruits of knowledge and science.
